# The Intriguing Sigma-1 and Sigma-2 Receptors and Their Potential Therapeutic Roles 2.0

**DOI:** 10.3390/ijms242115868

**Published:** 2023-11-01

**Authors:** Carmen Abate, Tangui Maurice

**Affiliations:** 1 Department of Pharmacy-Drug Sciences, University of Bari “A. Moro”, 70125 Bari, Italy; 2 MMDN, University of Montpellier, EPHE, INSERM, 34095 Montpellier, France

For some time now, the research on sigma receptors has been at a high level of maturity but, despite everything that has already been achieved, further work in this field still holds huge appeal, with vast possibilities for original discoveries. This, the second Special Issue of *International Journal of Molecular Sciences* covering the intriguing potential of sigma-1 and sigma-2 receptors and their possible therapeutic roles, particularly illustrates this view. The sigma-1 and sigma-2 proteins have been identified, their cellular functions and pharmacological roles are better understood, their involvement in pathologies and their potential as therapeutic targets have been better demonstrated, and the requirements for the design of effective and selective drugs or tools to finely modulate their activity are now well established [1,2,3,4,5,6,7,8]. The fifteen articles included in this collection provide a wide overview of the most urgent questions regarding sigma-1 and sigma-2 receptors in terms of their chemistry, pharmacology, and therapeutic indications. This Special Issue can be specifically seen as a forum, reporting the most recent developments and bridging the various important communications presented during the 3rd and 4th European Symposia on the Physiopathology of Sigma-1 Receptors, which were held, respectively, in October 2021 in Bari, Italy, and in September 2023 in Montpellier, France.

The chemistry of sigma-1 ligands has been comprehensively developed since the first proposal of a pharmacophore model by Glennon et al. in 1994 [9]. Recently, the focus has been on the molecular dynamics of ligand interaction with the sigma-1 protein in all its physical states. As an example of this, by combining bioinformatics analysis with S1R sequence and structural modelling approaches, Kim and Bezprozvanny [Contribution 1] provide novel mechanistic insights into the signaling functions of sigma-1 receptors in cells. They propose a model that suggests that S1R may exist in two distinct conformations, and could be considered as either a dynamic or anchored monomer, which may help to explain the neuroprotective effects of sigma-1 ligands. For their article, Pascarella et al. [Contribution 2] performed molecular dynamics simulations combined with computational and virtual screening procedures to help clarify the potential access route of ligands, and notably steroids, to the sigma-1 receptor binding site. They confidently confirm that ligands can access the binding site through a cavity that opens on the protein surface in contact with the membrane. Li et al. [Contribution 3] propose, in their study using intact retinal pigment epithelial cells, that sphingosine, or its derivative N,N′-dimethylsphingosine, readily binds the sigma-1 protein via a membrane bilayer pathway and, therefore, acts as an endogenous ligand controlling sigma-1 activity.

Three contributions illustrate the diversity of sigma-1 pharmacology. Firstly, Voronin et al. [Contribution 4] explore the hypothesis that the sigma-1 receptor is involved in GABA_A_ receptor-dependent pharmacological effects by showing that selective sigma-1 agonists and antagonists modulate the anticonvulsive effect of diazepam. Secondly, Oxombre et al. [Contribution 5] describe a novel benzamide-derived compound acting as a sigma-1 agonist with favorable ADME properties and the ability to significantly reduce clinical progression in a multiple sclerosis model, the experimental autoimmune encephalomyelitis in mice. Thirdly, Wilson et al. [Contribution 6] describe the antinociceptive and antiallodynic efficacy of SI 1/28, a recently reported benzylpiperazine derivative and, in particular, show that it appears to be effective in the treatment of acute inflammatory pain and chronic neuropathy, without liabilities, at therapeutic doses.

Neuroprotection, in diverse indications including chronic progressive neurodegenerative pathologies, remains a major outcome for sigma-1 agonists [2,3,5,7], and four articles in this collection offer new information on this topic. Lachance et al. [Contribution 7] provide a valuable review article describing the sigma-1 receptor subcellular localization and biological activity within specific subcellular compartments, namely, the mitochondria-associated endoplasmic reticulum (ER) membrane, the ER–lipid-droplet interface, the ER membrane interface, and the nuclear envelope. They discuss how the dysregulation of these inter-organelle pathways contributes to neurodegenerative diseases, while highlighting the cellular mechanisms and key binding partners engaged in these processes. On a different note, Voronin et al. [Contribution 8] contributed a meta-analysis of the mechanisms of ER stress and the unfolded protein response in the pathogenesis of several neurodegenerative diseases by compiling data on BiP and sigma-1 chaperones in clinical and experimental studies of Alzheimer’s disease, Parkinson’s disease, amyotrophic lateral sclerosis, and Huntington’s disease. The analysis strongly supports the feasibility of targeting pharmacological regulation of the chaperone function in neurodegenerative pathologies. Moreover, a concise and clear inventory of the cellular pathways involved in sigma-1-receptor-mediated cytoprotection is proposed by Couly et al. [Contribution 9]. Finally, to return to drug development, Gaja-Capdevila et al. [Contribution 10] describe two novel sigma-1 ligands. They used an in vitro model of spinal cord organotypic cultures under chronic excitotoxicity; and two in vivo models, spinal nerve injury and superoxide dismutase 1 (SOD1)^G93A^ mice; to analyze their effects on motoneuron survival and the modulation of glial reactivity. Their data show that the compound, acting as an agonist, is particularly efficient in preserving motoneurons from degeneration.

The sigma-2 receptor, identified more recently than the sigma-1 protein, is encoded by TMEM97 and expressed in the brain and retinal cells. Curiously, it is a completely different protein to sigma-1 but shares some pharmacological characteristics and also acts as a cell physiology modulator via protein–protein interactions, such as with progesterone receptor membrane component 1 (PGRMC1). Lizama et al. [Contribution 11] provide a complete review describing the key pathways that are modulated by sigma-2 receptors and are involved in age-related diseases, including autophagy, trafficking, oxidative stress, and amyloid-β and α-synuclein toxicity. The sigma-2 receptor, like the sigma-1 protein, is druggable and has the ability to be activated or inactivated by small molecules. Xu et al. [Contribution 12] present a novel series of sigma-2 compounds containing benzimidazolone and diazacycloalkane cores, which they characterized using radioligand binding assays and computational chemistry. The selective sigma-2 ligands are particularly valuable, as they allow the discovery of a novel mode of action by the receptor. A remarkable example is given in the study by Thejer et al. [Contribution 13], which shows that sigma-2 receptor ligand binding modulated the association between the translocator protein TSPO and TMEM97 in MCF7 breast adenocarcinoma and MIA PaCa-2 pancreatic carcinoma cells. Indeed, the sigma-2 receptor is overexpressed in proliferating tumor cells and may be a potentially valuable antiproliferative target. To further investigate the mechanism of action, Sorbi et al. [Contribution 14] designed and synthesized BS148, a fluorescent probe based on a sigma-1 modulator that penetrated SK-MEL-2 melanoma cells. They reported that BS148 was able to inhibit metastatic melanoma cell proliferation and migration through its interaction with the sigma-2 receptor, corroborating the position of the sigma-2 receptor as a promising target to treat cancer [6]. Finally, as both sigma-1 and sigma-2 receptors share common indications, although likely through very different mechanisms, Li et al. [Contribution 15] share a comparative study examining the differential responses to sigma-1 or sigma-2 receptor ablation in adiposity, fat oxidation, and sexual dimorphism in Sigma-1^−/−^ and Tmem97^−/−^ mice. Their data show that mechanistic investigations could lead to translational strategies to target differential sigma-1/sigma-2 regulations and sexual dimorphism for precision treatments for obesity.

As can be seen, the wide range of contributions presented in this Special Issue cover all aspects of the current research into sigma-1 and sigma-2 receptors and offer insights not only into the consideration of sigma-1- or sigma-2-related cell mechanisms in pathophysiological conditions but also into the pharmacological value of sigma-1- or sigma-2-based drug development in pathological indications.
